# Chromosome 10 abnormality predicts prognosis of neuroblastoma patients with bone marrow metastasis

**DOI:** 10.1186/s13052-021-01085-6

**Published:** 2021-06-09

**Authors:** Chi-yi Jiang, Xiao Xu, Bing-lin Jian, Xue Zhang, Zhi-xia Yue, Wei Guo, Xiao-li Ma

**Affiliations:** 1 Medical Oncology Department, Pediatric Oncology Center, Beijing Children’s Hospital, Capital Medical University, National Center for Children’s Health, Beijing Key Laboratory of Pediatric Hematology Oncology, Key Laboratory of Major Diseases in Children, Ministry of Education, Beijing, 100045 China; 2 MILS (Beijing) Medical Labortory, Beijing, China

**Keywords:** Chromosome 10, Neuroblastoma, Cancer survival, Chromosome G-banding

## Abstract

**Background:**

Neuroblastoma (NB) is the most common extracranial solid tumor in children. It is known for high heterogeneity and concealed onset. In recent years, the mechanism of its occurrence and development has been gradually revealed. The purpose of this study is to summarize the clinical characteristics of children with NB and abnormal chromosome 10, and to investigate the relationship between the number and structure of chromosome 10 abnormalities and NB prognosis.

**Methods:**

Chromosome G-banding was used at the time of diagnosis to evaluate the genetics of chromosomes in patients with NB and track their clinical characteristics and prognosis. All participants were diagnosed with NB in the Medical Oncology Department of the Beijing Children’s Hospital from May 2015 to December 2018 and were followed up with for at least 1 year.

**Results:**

Of all 150 patients with bone marrow metastases, 42 were clearly diagnosed with chromosomal abnormalities. Thirteen patients showed abnormalities in chromosome 10, and chromosome 10 was the most commonly missing chromosome. These 13 patients had higher LDH and lower OS and EFS than children with chromosomal abnormalities who did not have an abnormality in chromosome 10. Eight patients had both MYCN amplification and 1p36 deletion. Two patients had optic nerve damage and no vision, and one patient had left supraorbital metastases 5 months after treatment.

**Conclusions:**

The results indicated that chromosome 10 might be a new prognostic marker for NB. MYCN amplification and 1p36 deletion may be related to chromosome 10 abnormalities in NB. Additionally, NB patients with abnormal chromosome 10 were prone to orbital metastases.

## Background

Neuroblastoma (NB) is a malignant solid tumor of children originating in the adrenal medulla and sympathetic nervous system [1]. It is the widely diagnosed in children, with 8–10.2 cases of NB occurring per million children under 15 years of age [2]. It has high heterogeneity, hidden onset, and poor prognosis, with a 5-year survival rate of less than 40% in high-risk NB [3]. Only 1–2% of NB varieties have genetic predisposition, and most cases are sporadic [4]. However, the mechanism of occurrence, proliferation, and metastasis of NB is not clear. With the development of chromosome karyotype analysis, whole-genome sequencing, and proteomics, the relationship between chromosome abnormalities and apoptosis, differentiation, spontaneous regression, proliferation, and metastasis of NB tumor cells has gradually been revealed [5].

Chromosome 10 is one of 23 pairs of autosomes in humans, and contains about 135 million base pairs. It is possible that important genes related to the development of NB exist on chromosome 10. One of the tumor suppressor genes, PTEN (phosphatase and tensin homolog), is located at 10q23.3. It can influence the development of NB through the PI3K/ AKT/ mTOR pathway [6]. Children with high-risk NB at Stage IV, without MYCN gene amplification, and with a whole chromosome aneuploidies (WCAS) factor of less than 2 have a poorer prognosis than those with a WCAS factor of greater than 2. This phenomenon is most significant on chromosome 10 (*P* = 0.002) [7]. A previous clinical study demonstrated that 50% of the children with metastatic NB had chromosomal abnormalities, and 70% of them have concurrent abnormalities related to chromosome number and structure. Among them, abnormalities in number occurred frequently on chromosomes 21, 10, and 11, with abnormalities on chromosome 10 being the most frequent.

This study aims to summarize the clinical characteristics of children with NB with abnormal chromosome 10 in a single treatment center. The relationship between the abnormal number and structure of chromosome 10 and the occurrence, development and prognosis of NB will be explored in order to to provide a new argument for chromosome genetics of neuroblastomas.

## Methods

### Patients samples

This study analyzed a total of 150 children with NB who had been diagnosed with bone marrow metastases by routine bone marrow cytology or bone marrow biopsy from May 2015 to December 2018 in the Medical Oncology Department of the Beijing Children’s Hospital. All patients were staged according to the International Neuroblastoma Stage System (INSS) [8]. Risk stratification was conducted according to the Children’s Oncology Group (COG) [9]. Bone marrow specimens were tested by chromosome G-banding when the patients were first hospitalized. All patients were regularly treated and followed up with at the center until November 30, 2019.

Complete medical records were collected for each participant. Clinical data included age at diagnosis, staging, sex, MYCN gene, chromosome report, and outcome. Based on detailed karyotype analysis, it was found that 13 patients had abnormal chromosome 10 and shared similar clinical characteristics. A focus was placed on their clinical features, such as primary tumor location, tumor markers at initial diagnosis, the largest diameter of the tumor, metastatic site, and so on. The study was approved by the Ethics Committee of the Beijing Children’s Hospital of Capital Medical University (2019-k-390). Informed consent was acquired from all participants and their parents prior to the collection of samples and information.

### Therapeutic regimen and follow-up

All patients were treated according to NB protocols of the Beijing Children’s Hospital (BCH-NB-2007), which was developed based on Hong Kong NB protocol 7 and the European low- and intermediate-risk (IR) NB protocol [10–12]. For the low-risk (LR) and IR groups with favorable pathological NB, therapies were CBVP (carboplatin, etoposide) and CADO (cyclophosphamide, doxorubicin, vincristine) alternately for 4–6 courses, and surgery. For the IR group with unfavorable pathological NB, therapy included 6–8 courses of chemotherapy combined with surgery, radiotherapy, and 6 courses of cis-retinoic acid. For the high-risk (HR) group, the chemotherapy regimen was CAV (cyclophosphamide, doxorubicin, vincristine) and CVP (etoposide, cisplatin), administered sequentially, which was combined with surgery, autologous hematopoietic stem cell transplantation, local radiotherapy, and 13-cis-retinoid acid. Patients were regularly followed up with every 3 months in the first year, every 4 months in the second year, and every 6 months in year 3 and 4.

### Chromosome examination

Chromosome karyotype analysis of bone marrow cells was performed on G-banded preparations. All methods were performed in accordance with the experiment guidelines and ethical permission. First, bone marrow specimens were anticoagulated with 2 ml heparin and cultured in 1640 culture media at 37 °C and 5.0% CO_2_ for 24 h. After adding 0.075 mol/L KCL hypotonic solution, 1.5 ml fixing solution (3,1 mixture of methanol and acetic acid) was used to mix and pre-fix two times [13]. Each specimen was divided into four pieces and baked at 80 °C overnight. Before the examination, each piece was digested with trypsin and stained with Giemsa. Each split phase was observed and analyzed using high resolution chromosome analysis system (SRL, Tokyo, Japan). Twenty metaphase mitotic phases were analyzed in each specimen.

The karyotypes were defined based on the International System for Human Cytogenomic Nomenclature (ISCN 2016) [14]. In tumor cells, it was considered a meaningful clone if 2 or more cells showed the same gain or structural abnormality of chromosomes, and 3 or more cells showed the same loss.

### Statistical analysis

Descriptive statistics were conducted using Statistical Package for Social Scientists (SPSS) version 22. Overall survival (OS) was defined as the time from enrollment to disease-caused death or final follow-up. Event-free survival (EFS) was defined as the time to first occurrence of any event, such as disease progression, or death from any cause. The survival curves for OS or EFS were generated via the Kaplan-Meier method, and the difference between the two groups was evaluated with a log-rank test. Correlation between chromosome 10 and MYCN gene was analyzed by continuous correction chi-square test, where *p* < 0.05 was considered statistically significant.

## Results

### Patient cohort

All 150 children with NB had bone marrow metastasis, and all of them underwent chromosome examination. According to the results of karyotype analysis, 42 (28%) cases exhibited chromosomal abnormalities, and 108 (72%) were normal. As shown in Table 1, age at diagnosis in the abnormal group ranged from 8 to 105 months (mean, 38 months). In terms of tumor markers, the median lactate dehydrogenase (LDH) and median neuron specific enolase (NSE) of the abnormal group were 1088 (range, 655, 3423.75) U/L and 370 (range, 364.33, 370) ng/l, respectively. In the normal group, the median LDH was 661 (range, 389.25, 1103.5) U/L, and the median NSE was 290.25 (range, 129.225, 370) ng/l.

**Table 1:** Characteristics at diagnosis of 150 NB patients

Characteristic	Chromosome normal	Chromosome abnormalities
Sex		
Male	61	25
Female	47	17
Age (months)		
<18	22	2
≥18	86	40
Staging		
IV	106	42
IVs	2	0
Risk group		
LR	3	0
IR	14	0
HR	91	42
MYCN		
Amplification	10	18
Not amplification	98	24
LDH (U/L)		
≤295	15	0
295–500	26	2
500–1500	53	23
>1500	14	17
NSE (ng/l)		
≤25	5	1
25–100	16	2
>100	87	39
Primary tumor site		
Retroperitoneum and adrenal glands	95	36
Mediastinum	11	5
Pelvic cavity	1	1
Neck	1	0

*LR* low-risk, *IR* intermediate-risk, *HR* high-risk, *MYCN* amplification of the MYCN gene, *LDH* lactate dehydrogenase, *NSE* neuron specific enolase

### Chromosome karyotype analysis

**Fig. 1:**
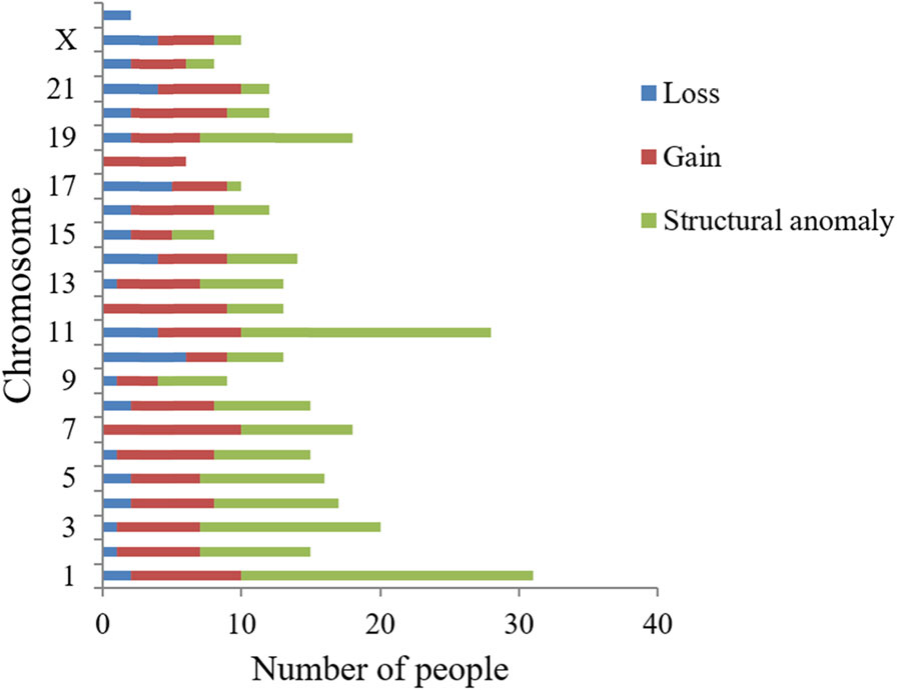
Distribution of abnormal number of chromosomes

Among the 42 children with definite chromosomal abnormalities, the number of chromosome losses was more than the number of chromosome gains (20 vs 16; 47.6% vs 38.1%). However, there were mainly gains in the number of detailed chromosome changes, as shown in Fig. 1. There were 29 patients with an abnormal number of chromosomes, including one with only gains of marked chromosomes. Thirteen patients displayed only chromosome losses, and 6 patients displayed only gains, while 9 patients had both gains and losses at the same time. The most frequent quantity anomalies were chromosome 10 losses (30%), chromosome 17 losses (25%), chromosome 7 gains (62.5%), and chromosome 12 gains (56.25%). Chromosome karyotype analysis showed that individual chromosomes differed in their likelihood of being lost or gained. For example, gains appeared frequently in chromosomes 1, 6, 7, 12, and 20. Losses were seem more commonly in chromosomea 10, 11, 14, 17, 21, and X. The karyotype of tumor patients was so complex that it was difficult to evaluate only in terms of abnormal quantity. Therefore, most of the above children (97.6%) also had abnormal chromosome structure.

### Correlation analysis of chromosome 10 anomalies

**Fig. 2:**
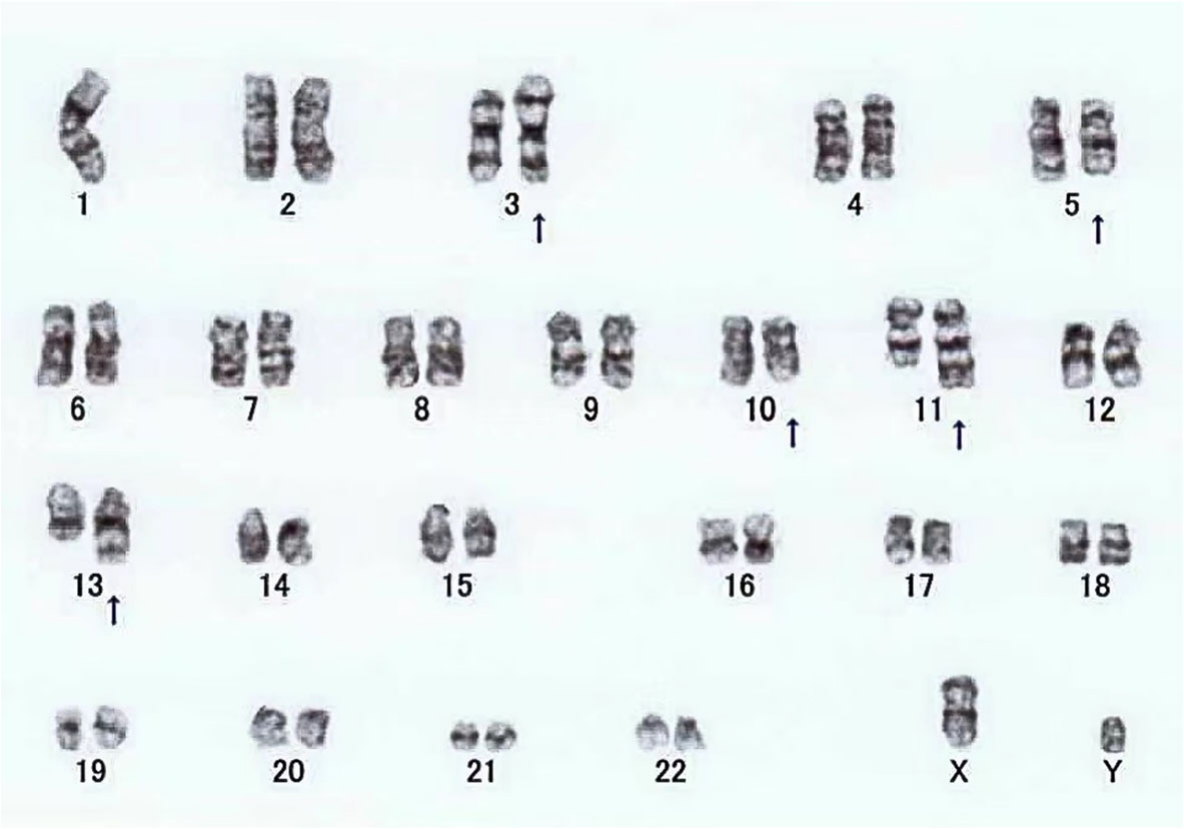
Chromosome G-banding karyotype image of a patient with 10q22 structural abnormality

A total of 13 children displayed chromosome 10 abnormalities, including 6 with chromosome 10 losses (46.2%), 3 with chromosome 10 gains (23.1%), and 4 with structural abnormalities (30.8%). All 13 were HR children with NB at stage IV (Table 2). As shown in Fig. 1, the loss of chromosome 10 was the most common among decreases in the number of chromosomes. All 4 patients with structural abnormalities had changes in the 10q22 area. One example of a karyotype image with 10q22 regional distortion is shown in Fig. 2. Of these children with chromosome 10 abnormalities, 10 patients (76.9%) had MYCN amplification. Analysis of chi-square testing showed that chromosome 10 abnormality was associated with MYCN amplification (χ2 = 41.706, *P <* 0.01). These cases were all accompanied by 1p36 deletion except for 2 cases without 1p36 examination. The median LDH and NSE of the chromosome 10 abnormal group at first diagnosis were 2833 (1073, 3743.5) U/L and 370 (370, 445) ng/l, respectively. Their LDH were higher than that of children with chromosome abnormalities who did not have a chromosome 10 abnormality (Mean, 968, 652, 2041.5 U/L), although the difference was not statistically significant (*P* = 0.185). The median vanillylmandelic acid (VMA) in the group with abnormal chromosome 10 was 30.34 (10.25, 183.01). Because the partial maximum of the NSE reported by the study center only shows > 370, the NSE of the two groups cannot be compared. Two children had optic nerve damage and no vision, while another child had left supraorbital metastases 5 months after treatment.

**Table 2:** Characteristics of NB with chromosome 10 abnormality

Characteristic	N	%
chromosome 10		
Gain	3	23.1
Loss	6	46.2
Structural anomaly	4	30.8
Sex		
Male	6	46.2
Female	7	53.8
MYCN		
Amplification	10	76.9
Not amplification	3	23.1
1p36		
Lost	8	61.5
Not lost	3	23.1
None	2	15.4
LDH (U/L)		
≤295	0	0
295–500	3	23.1
500–1500	0	0
>1500	10	76.9
NSE (ng/l)		
≤25	1	7.7
25–100	0	0
>100	12	92.3
VMA (mg/24 h urine)		
≤13.6	5	38.5
>13.6	7	53.8
None	1	7.7
Primary tumor size		
≤5	0	0
5–10	4	30.8
>10	8	61.5
Metastasis site		
Bone	10	76.9
Bone marrow	13	100
Harnpan/Intracalvarium	9	69.2
Periorbital	5	38.5
Mediastinum	4	30.8
Lymph nodes	11	84.6
Other visceral organ	5	38.5

MYCN amplification of the MYCN gene, 1p36 loss of 1p36, LDH lactate dehydrogenase, *NSE* Neuron specific enolase, *VMA* Vanillylmandelic acid

### Outcome and prognosis

**Fig. 3:**
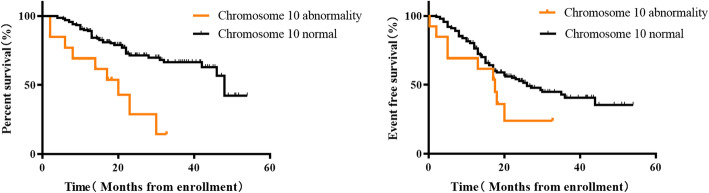
Kaplan-Meier estimates of survival in 150 patients with NB according to the status of chromosome 10 **a** Overall survival for all patients. **b** Event-free survival for all patients

**Fig. 4:**
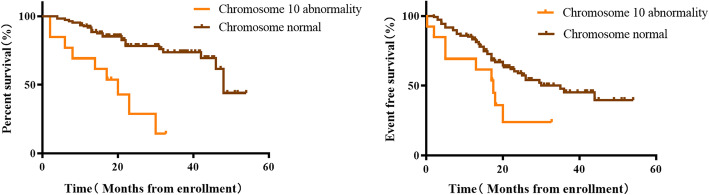
Kaplan-Meier estimates of survival in 110 patients with NB according to the status of chromosome 10 **a** Overall survival for all patients. **b** Event-free survival for all patients

**Fig. 5:**
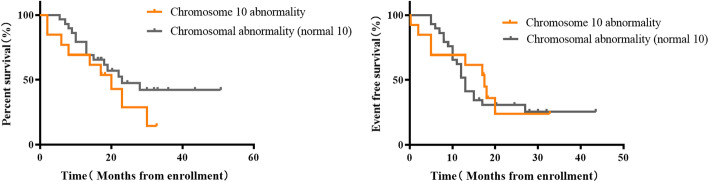
Kaplan-Meier estimates of survival in 42 patients with NB who had chromosome abnormalities according to the status of chromosome 10 **a** Overall survival for all patients. **b** Event-free survival for all patients

After definite diagnosis, the children were treated and followed up according to risk stratification. Among the 13 patients with a definite chromosome 10 abnormality, whose median follow-up was 17.25 (7, 21.5) months, 9 had recurrences. The median progression time was 13 (3.5, 17.75) months. All 9 children died. Thirteen NB patients with abnormal chromosome 10 had significantly lower three-year OS than 143 patients with normal chromosome 10 (66.37% vs 14.36%, *P* = 0.002). However, the three-year EFS was lower in the abnormal chromosome 10 group, but this was not statistically significant (40.62% vs 23.93%, *P* = 0.0837, Fig. 3). Three-year OS was 14.36% in the group with abnormal chromosome 10 versus 73.63% in the normal group. The three-year EFS of the group with abnormal chromosome 10 was also higher than the normal group (23.93% vs 45.27%). There were statistical differences between the above two groups (*P* < 0.001; *P* = 0.0179, Fig. 4). The three-year OS and EFS of the group with chromosome abnormalities without abnormal chromosome 10 were 42.25 and 25.54% respectively. As shown in Fig. 5, the OS of the group with chromosome abnormalities without abnormal 10 was higher than the group with abnormal chromosome 10, although this was not statistically significant (*P* = 0.2158; *P* = 0.7817).

## Discussion

As a heterogeneous and occult tumor, most children with NB have chromosomal abnormalities when they are first diagnosed [15]. According to the Mitelman database, more than 60% of NB is aneuploidy [16]. In WCAS, NB is prone to co-occur with trisomy of chromosomes 6–9, 12, 13, 17, 18, 20, and 21, and monosomy of chromosomes 3, 4, 9–11, 15, 17, 19, 22 and X [7]. NB in stages I, II, and 4S is mostly triploid, with relatively good prognosis. Patients often have chromosome 6, 7, and 17 gains and chromosomes 3, 4, 11, and 14 losses [17]. Parodi et al. came to a preliminary conclusion that the prognosis of children with NB and whole X-chromosome-loss is relatively poor, which can be used as a new prognostic indicator, and patients with this chromosomal abnormality should be treated in the IR group [18]. Marked by the centromere, each chromosome is divided into a long arm (q) and short arm (p). High-risk NB children without MYCN gene amplification at stage IV often show an increase in the number of chromosomes 7, 12 and 17, lost of 11q and 3p alleles, and 17q gains [19]. However, no specific association between chromosome 10 and neuroblastoma has been reported. As can be seen from Fig. 1, in accordance with previous studies, the most common gain is seen in chromosome 7, and the most common structural abnormalities are seen in chromosomes 1 and 11. Chromosome 10 is the most frequent loss.

Previous studies have shown that there are tumor suppressor genes such as PTEN, DBMT, and LGI1 on the long arm of chromosome 10, and IDI1, AKR1C3, DDH1, NET1A, PRKCQ, and GATA-binding protein 3 on the short arm [20, 21]. Among them, PTEN is the second largest deletion/mutation gene in human tumors, with a mutation rate of 50% [22]. Li et al. [23] have confirmed that GDNF family receptor alpha 2(GFRA2) promotes proliferation of NB cells by activating the PTEN/PI3K/AKT pathway. The allele imbalance of 10p 11.23–15.1 and 8q 21.3 appears to be specific to stage 4 tumors with MCYN amplification [24]. The complete loss of chromosome 10 is common in tumors of the brain, lungs, ovaries, and skin [25]. Although there are no prior studies of chromosome 10 and NB, it has been reported that genetic changes in chromosome 10q are common in other neurological tumors [26]. For example, members of the cysteine-rich scavenger receptor family, DMBT1(10q25.3–26.1), are heterozygously absent in oligodendrogliomas, medulloblastoma, gastrointestinal cancer, and lung cancer [27, 28]. Park et al. reported that in the CpG island methylator phenotype (G-CIMP) subtype of glioma, children with 10q loss have a poor prognosis [29]. MGMT, located at 10q26.1, encodes a protein associated with DNA repair that can remove proto-mutant alkyl from O. It affects the development of glioblastoma by the oncogene TP53 [30]. Fibroblast growth factor receptor 2 (FGFR2) at 10q26 is associated with cell proliferation, differentiation, migration, and inhibition of apoptosis. It is overexpressed in breast cancer [31], and downregulated in prostate cancer [32].

This study showed that the OS rate of NB children with abnormal chromosome 10 was significantly lower than that of children with normal chromosome 10, including children with no chromosomal abnormalities and children with abnormal chromosomes but normal chromosome 10. The OS and EFS of the group with all normal chromosomes were significantly higher than those in the abnormal chromosome 10 group. 10q22 was found to be the sites of all structural abnormalities on chromosome 10, which indicates that 10q22 may have tumor suppressor or oncogenic genes. Other non-statistically significant results may be due to the number of chromosome 10 abnormalities still being insufficient.

In addition, the individual effects of chromosome 10 abnormalities cannot be evaluated because many abnormal chromosome karyotypes in children with NB contain complex quantitative or structural abnormalities. There may be other chromosomes associated with neuroblastoma in the group of chromosomal abnormalities affecting prognosis.

## Conclusions

In summary, although it was only a preliminary effort, this study demonstrated that chromosome 10 might be used as a new prognostic marker for NB with bone marrow metastasis. NB children with abnormal chromosome 10 were prone to MYCN amplification and 1p36 deletion, and their outcomes were worse. MYCN amplification which occurs simultaneously with deletion of 1p36 may be related to chromosome 10 abnormalities in neuroblastoma. Additionally, the NB cells of these children were easy to metastasize to orbit. 10q22 may be the site on chromosome 10 that is associated with NB proliferation and metastasis. For future studies, the sample size will be expanded, focusing on 10q22 changes and 11q23 deletion.

## Data Availability

Key data generated or analyzed during this study are included in this published article. Any additional dataset is available from the corresponding author upon reasonable request.

## Abbreviations

NB: Neuroblastoma
WCAS: Whole chromosome aneuploidies
INSS: International Neuroblastoma Stage System
COG: Children’s Oncology Group
IR: Intermediate-risk
LR: Low-risk
HR: High-risk
ISCN: International System for Human Cytogenomic Nomenclature
SPSS: Statistical Package for Social Scientists
OS: Overall survival
EFS: Event-free survival
LDH: Lactate dehydrogenase
NSE: Neuron specific enolase
VMA: Vanillylmandelic acid
FGFR2: Fibroblast growth factor receptor 2
